# Mortality from suicide among adolescents and young people in the Altai Republic, Russia, for the period of 1990-2019

**DOI:** 10.1192/j.eurpsy.2022.1513

**Published:** 2022-09-01

**Authors:** N. Semenova

**Affiliations:** Scientific Research Institute of Medical Problems of the North, Department Of Child’s Physical And Mental Health, Krasnoyarsk, Russian Federation

**Keywords:** Altaians, Adolescents, Suicide, Epidemiology

## Abstract

**Introduction:**

The Altai Republic (AR) is the national subject of the Russian Federation where suicidal situation is unfavorable as the death rate from suicide exceeds the national rates by three times. The high level of suicide among adolescents and young people is especially alarming.

**Objectives:**

To analyze the dynamics of mortality from suicide among adolescents and young people in the AR for the period from 1990 to 2019.

**Methods:**

Data on mortality of the population were obtained from the Russian databases of demographic indicators and analyzed in terms standardized per 100,000 population.

**Results:**

The highest mortality rates from suicide are recorded in the 20-24 age group. The suicide rate, compared to 1990, decreased slightly from 82.5 (in 1990) to 79.7 per 100 thousand (in 2019). The dynamics of mortality from suicide among adolescents aged 15-19 is also unstable. When comparing the indicators in 1990 and in 2019, one can verify the negative dynamics in the form of a 4-fold increase in the suicide rate – from 14.4 (in 1990) to 58 (in 2019). An analysis of the ethnicity of young people who committed complete suicide showed that the majority of suicides (90%) were indigenous Altaians.

**Figure:**
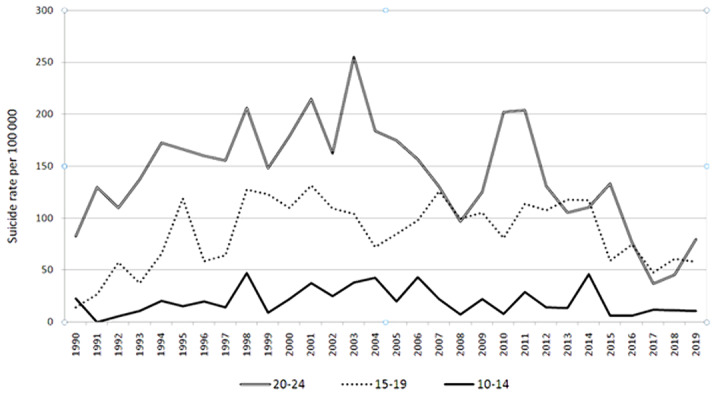

**Conclusions:**

In adolescents and young people of the AR, the death rate from suicide exceeds the all-Russian indicators from 7.9 to 9.3 times. Effective preventive measures are needed to improve the situation.

**Disclosure:**

No significant relationships.

